# Identification and validation of biomarkers related to mismatch repair for prognosis prediction in glioma

**DOI:** 10.3389/fimmu.2026.1652739

**Published:** 2026-06-23

**Authors:** Jia Feng, Yuankai Si, Long Han, Yilan Huang, Longyang Jiang

**Affiliations:** 1 Department of Pharmacy, The Affiliated Hospital of Southwest Medical University, Luzhou, China; 2 Department of Pharmacy, Southwest Medical University, Luzhou, China; 3 Department of Pharmacy, Qingdao University Affiliated Women and Children’s Hospital, Qingdao, China; 4 Department of Pharmacy, Peking University People’s Hospital Qingdao Hospital, Qingdao, China

**Keywords:** bioinformatics, drug sensitivity, glioma, mismatch repair-related genes, prognostic model

## Abstract

**Background:**

Gliomas are aggressive brain tumors with a dismal prognosis, and their development, progression, and responsiveness to treatment are all impacted by mismatch repair (MMR) deficiency. This work aimed to construct a predictive risk model based on MMR-related genes and confirm its clinical applicability, with a focus on identifying novel therapeutic targets.

**Methods:**

Clinical and mRNA expression data from The Cancer Genome Atlas (TCGA) and The Chinese Glioma Genome Atlas (CGGA) glioma patients were analyzed. MMR-related genes were sourced from the Molecular Signatures Database (MSigDB). A risk score model was created using multivariate Cox regression and LASSO analysis. Patients were categorized into high- and low-risk groups based on the median risk score. The model’s performance was assessed using ROC curves, AUC, and Kaplan-Meier survival analysis. Immune cell infiltration was quantified using “CIBERSORT” and “QUANTISEQ”. Immunotherapy potential was evaluated via TMB, TME, and TIDE scores. The half maximal inhibitory concentration (IC_50_) analyses were performed. *In vitro* validation involved MCM8 overexpression/knockdown in U251/LN229 glioma cells, followed by TMZ sensitivity testing via CCK-8 assays.

**Results:**

An eight-MMR-related gene prognostic model (HMGB1, MCM8, MUTYH, PMS1, RNASEH2B, RNASEH2C, RPA3, TP73) was constructed. The risk score was an independent prognostic factor, with high-risk patients showing significantly poorer overall survival. The validity of this model has been validated in the CGGA dataset. Significant differences in immune infiltration, TME, and TMB scores were observed between risk groups. Drug sensitivity analysis revealed distinct IC_50_ profiles for chemotherapeutic agents between the groups. Most importantly, *in vitro* experiments demonstrated that MCM8 overexpression increased glioma cell sensitivity to TMZ, while MCM8 knockdown decreased it, identifying MCM8 as a potential target for TMZ-based personalized therapy.

**Conclusion:**

This study establishes a robust MMR-related prognostic model for glioma that effectively stratifies patients, predicts survival, and reflects distinct immune microenvironments. Critically, we identify MCM8 as a novel and actionable biomarker that modulates TMZ sensitivity, offering a promising avenue for personalized treatment in glioma patients.

## 1 Introduction

The Global Cancer Statistics Report 2022 states that there were 248,305 new fatalities and 321,476 new cases of brain and nervous system malignancies globally (1). Glioma, as a neuroepithelial brain tumor originating from glial cells, is an intracranial malignant tumor and accounts for approximately 81% of primary malignant tumors of the central nervous system (2). Conventional treatment of gliomas usually includes surgery, chemotherapy and radiotherapy (3). However, glioma cells possess biological complexity, like heterogeneity, high proliferation rate, and infiltrative characteristics, resulting in a high recurrence rate and susceptibility to drug resistance. It is considered to be one of the most devastating cancers due to its low 5-year survival rate, high mortality rate, high disability rate, and high recurrence rate (4, 5), which seriously threaten patients’ lives and affect their quality of life. Therefore, the treatment of glioma has become a research hotspot in neuro-oncology.

Despite the increasing understanding of the pathological mechanisms of glioma, the prognostic quality of patients remains poor (6). As molecular pathology research has advanced in recent years, biomarkers such IDH, TP53, EGFR, CDKN2A, MGMT, and others have become increasingly important in the diagnosis and management of gliomas (7). Currently, research has identified several biomarkers that can be used to predict survival rates in patients with glioma, such as m5C-associated factors (8), immune-related genes (2), dephosphorylation-related genes (9). Nevertheless, glioma treatment is still not as effective as it should be, and the prognosis of patients is not optimistic. Therefore, it has become imperative to explore new reliable biomarkers and explore prognostic indicators for glioma patients.

The Deoxyribonucleic Acid (DNA) mismatch repair system is considered to be the main genetic mechanism for stabilizing the structure of DNA and maintaining its function (10), and is mainly responsible for correcting DNA base insertion/deletion errors and base mismatches involved in mitotic and meiotic recombination, apoptosis, immunoglobulin gene rearrangements, somatic hypermutation, and other processes (11). By correcting DNA mismatches, mutations and tumors can be prevented in the short and long term, respectively (12). In carcinogenesis, mismatch repair genes are the third most genetically mutated genes besides oncogenes and tumor suppressor gene.

Several studies have found that mismatch repair is closely related to the tumor microenvironment in gastric, colorectal, and endometrial cancers (13–15). Currently, mismatch repair (MMR) deficiency is a key biomarker for predicting immune checkpoint blockade response in colorectal, endometrial and other tumors (16–18). In recent years, studies on the prognostic value of MMR-related genes in a variety of cancers have gradually increased. However, there is still a gap in research on gliomas. Notably, emerging evidence indicates that MMR alterations are clinically relevant in glioma. Caccese et al. demonstrated that MMR protein expression alterations occur more frequently in grade III gliomas, recurrent cases, TMZ-treated patients, and IDH-mutant gliomas (19). Furthermore, Suwala et al. identified a distinct aggressive subtype-primary mismatch repair-deficient IDH-mutant astrocytoma (PMMRDIA) -which universally exhibits MMR defects and carries the poorest prognosis among IDH-mutant gliomas (20). These findings underscore the potential clinical significance of MMR status in glioma but also highlight the need for a systematic evaluation of MMR-related gene signatures for prognostic prediction and therapeutic guidance. Temozolomide (TMZ) is one of the most widely used chemotherapy regimens for gliomas (21), and it has been demonstrated to increase survival in patients with recently diagnosed gliomas (22). Nevertheless, TMZ resistance remains a challenge for glioma chemotherapy. It has been found that deletion of the MMR pathway often produces TMZ resistance.

In the broader landscape of multi-omics and pathway-based biomarker discovery, integrative approaches have successfully identified oncogenic determinants across tumor types by systematically linking genomic instability, immune microenvironment features, and therapeutic response (23). Nevertheless, the prognostic and therapeutic implications of mismatch repair (MMR)-related genes in glioma have not been systematically evaluated within such an integrative framework. Therefore, in this study, we aimed to comprehensively analyze the effects of MMR-related genes on the survival and prognosis of glioma patients from a bioinformatics perspective and construct a prognostic risk assessment model. To analyze the heterogeneity of immune microenvironment, chemotherapy sensitivity and immunotherapy response among patients with different risks. We also verified the effect of mini chromosome maintenance 8 homologous recombination repair factor (MCM8) on TMZ resistance, hoping to provide new research direction and reference for the individualized treatment of glioma patients.

## 2 Methods

### 2.1 Data collection

The Cancer Genome Atlas (TCGA) database and The Chinese Glioma Genome Atlas (CGGA) database were used to download all raw data, including glioma transcriptome data (FPKM and counts) and clinical data of glioma patients, such as survival time, survival status, age, gender, and new events, and cancer status. For further investigation, the mRNA expression data in FPKM format were subsequently converted to TPM format. Furthermore, 38 MMRs were chosen from the Molecular Signatures Database (MSigDB). 33 MMRs were obtained as candidate genes for further study after 5 genes that were not included in the TCGA database were eliminated.

### 2.2 Survival analysis and construction of MMR-based prognostic risk model

Gene expression data and clinical data were utilized to screen variables using Least Absolute Shrinkage and Selection Operator (LASSO) regression to avoid overfitting. Then, multivariate survival analysis was combined with Cox proportional risk regression model to build a risk model with the key genes, and risk scores were calculated for each glioma patient from the TCGA-glioma dataset. LASSO regression was employed for gene selection due to its advantage in handling high-dimensional data through L1 regularization, which reduces overfitting by shrinking less relevant coefficients to zero. Multivariate Cox proportional hazards regression was subsequently applied to construct the risk model, as it is the standard method for analyzing time-to-event survival data and allows for the assessment of independent prognostic effects while adjusting for potential confounders. This combined approach is widely recognized for developing robust prognostic signatures in cancer genomics. The multivariate Cox proportional hazards model coefficients were multiplied by the gene expression levels of each patient to determine the risk score. $\text{Risk score}=\sum_{i}^{n}Coef\left(i\right)X\left(i\right)$ (Coef: coefficients, X: transcription level). The patients with glioma were classified into low-risk and high-risk groups according to their median risk scores. The survival disparities between the two risk groups were examined using the log-rank test and Kaplan-Meier survival analysis. The Area Under Curve (AUC) values were computed using time-dependent subject Receiver Operating Characteristic (ROC) curves, and the “survival ROC” in R software was used to evaluate the risk model’s prognostic performance. The CGGA dataset was used as an external validation dataset to evaluate the model’s predictive performance.

### 2.3 Gene alteration and expression analysis in risk models

A genetic variation analysis of selected genes in the prognostic risk model was performed using the cBioPortal database. We used the “ComplexHeatmap” and “Vioplot” packages in the R software to create waterfall and violin plots and counted the frequency of mutations by mutation type and mutated gene. 8 MMR-related genes were also plotted in heatmaps to assess whether there were any differences in expression between high- and low-risk populations.

### 2.4 Risk score correlations with clinical parameters

Traditional clinical characteristics, such as age, gender, stage, cancer status, Tumor Node Metastasis (TNM) stage, and new events were included together with risk scores as probable independent prognostic factors to ascertain whether risk scores are an independent prognostic factor. Cox regression (univariate and multivariate) was performed to evaluate the relationship between these parameters and patient prognosis.

### 2.5 Tumor microenvironment and immune cell infiltration analysis

To examine the connection between risk scores and immune cell infiltration, two algorithms, “CIBERSORT” and “QUANTISEQ” were utilized to measure the degree of immune cell infiltration in the two groups. In the meantime, the “ESTIMATE” R software program was used to assess the Stromal Score, Immune Score, and ESTIMATE Score of the two groups to demonstrate the connection between the tumor microenvironment and the risk score.

### 2.6 Immune therapy reactions and chemical sensitivity analysis

Immunotherapy response data for gliomas were obtained from the Tumor Immune Dysfunction and Exclusion (TIDE) web portal (http://tide.dfci.harvard.edu/). Using this resource, researchers computed TIDE scores to evaluate how effectively patients in the high-risk and low-risk categories could circumvent tumor immune responses. For both patient subgroups, the Tumor Microenvironment (TME) score was determined through the “TMEscore” package in R. Additionally, somatic mutation profiles were leveraged to calculate the Tumor Mutation Burden (TMB) score for every individual with glioma across these risk-stratified groups. Seven potential chemotherapeutic agents for glioma including TMZ, imatinib, bortezomib, erlotinib, tamoxifen, vincristine and etoposide were screened from the Genomics of Drug Sensitivity in Cancer (GDSC) database. Leveraging the “pRRophetic” R package, we evaluated the half maximal inhibitory concentration (IC_50_) values of these drugs in glioma patients stratified into high- and low-risk groups. This analysis aimed to uncover potential links between risk scores and drug responsiveness. Additionally, score modeling was then correlated between the candidate chemotherapeutic agents and the eight genes used to construct the risk score model.

### 2.7 Cell culture

To further investigate the relationship between the MCM8 gene and TMZ drug sensitivity, we performed gene overexpression and knockdown experiments using U251 and Ln229 cells. The cell cultures were grown in a nutrient-rich medium supplemented with 10% fetal bovine serum and a 1% antibiotic cocktail (penicillin-streptomycin). These cultures were maintained under controlled conditions in a specialized incubator, set at 37 °C with a 5% carbon dioxide atmosphere to mimic physiological growth conditions.

### 2.8 Verification of transfection model

Overexpression plasmids and siRNA interference fragments were both transfected using Lipo3000 transfection reagent according to the instructions, respectively. The cells were collected 48 hours after transfection, and the MCM8 mRNA expression level was quantified by qRT-PCR to verify the efficiency of the model construction. The primers for MCM8 and β-actin were shown in **Table 1**. To ensure consistent transfection efficiency across experiments, the following optimization and control measures were implemented. First, preliminary experiments were conducted to optimize transfection conditions, including the ratio of transfection reagent (Lipo3000) to plasmid/siRNA (1:1 to 1:3, v/w), cell seeding density (70-80% confluence at the time of transfection), and incubation time (48 hours post-transfection). Second, a non-targeting negative control (NC) was included in all transfection experiments for both overexpression and knockdown groups to account for non-specific effects. Third, transfection efficiency was quantitatively validated by qRT-PCR, with predefined acceptance criteria of ≥70% reduction in mRNA expression for siRNA-mediated knockdown and ≥2-fold increase for overexpression compared to the NC group. Only experiments meeting these criteria were included in subsequent analyses. All transfection experiments were performed in three independent biological replicates, each with three technical replicates. Moreover, whole-cell protein extracts were obtained using RIPA lysis buffer. Following preparation of SDS-PAGE gels, electrophoretic separation was carried out. The resulting samples were then subjected to overnight incubation with primary antibodies targeting both MCM8 and β-actin proteins. The samples were then incubated with MCM8 antibody and β-actin internal reference antibody at 4 °C overnight. The following day, the samples were treated with corresponding dilutions of the secondary antibody and left to incubate at ambient temperature for two hours. To complete the process, chemiluminescent detection was performed using ECL reagent, followed by grayscale analysis with ImageJ 2.0 software.

**Table 1:** Primer sequences.

Gene	Forward	Reverse
MCM8	GCTCTCCTCTCACAGTTA CGATGG	GTGGAATCCGACCTGC TTCTCTC
β-actin	TGTGTCCGTCGTGGATCTGA	CCTGCTTCACCACCTTCTTGA

### 2.9 Measurement of IC_50_

Cells from the logarithmic growth phase of U251 and LN229 were collected, seeded at a density of 5×10^3^ cells per well in a 96-well plate, and transfected. The cells were incubated in a culture incubator for 48 hours until confluence reached 70–80%. TMZ was first dissolved in dimethyl sulfoxide (DMSO), followed by serial dilution using complete medium containing 10% fetal bovine serum (FBS) to obtain a working solution with a gradient concentration of 0, 0.3, 0.6, 1.2, 2.4, 4.8, 9.6, 19.2, 38.4, 76.8μg/mL. Set up three replicate wells for each drug concentration. Add 100μL of the corresponding drug solution to each well, while the blank control group was added to an equal volume of complete medium. Set up three replicate wells for each drug concentration. Add 100μL of the corresponding drug solution to each well. Add an equal volume of complete medium to the blank control group. After 48hours of drug treatment, add the CCK-8 reagent to each well and keep it for another hour. Finally, the absorbance values of each well were measured at 450nm using a microplate reader. The data obtained were analyzed using GraphPad Prism 9.0 software for dose-response curve fitting, and the IC_50_ values were calculated.

### 2.10 Statistical analysis

The Perl programming language was used to process data. R program (version 4.0.3) and its companion package were used to conduct statistical analysis and create graphs. GraphPad (Prism 9) software was used to process and analyze the experimental data. All *in vitro* experiments were performed as three independent biological replicates, each with three technical replicates. Data are presented as mean ± standard deviation (SD). The consistency of results across replicates was assessed by calculating the coefficient of variation (CV), and a CV< 15% was considered acceptable. The t-test was used to compare group differences. Statistical significance was established at *p* < 0.05.

## 3 Results

### 3.1 MMR-related prognostic gene screening results

**Figure 1:**
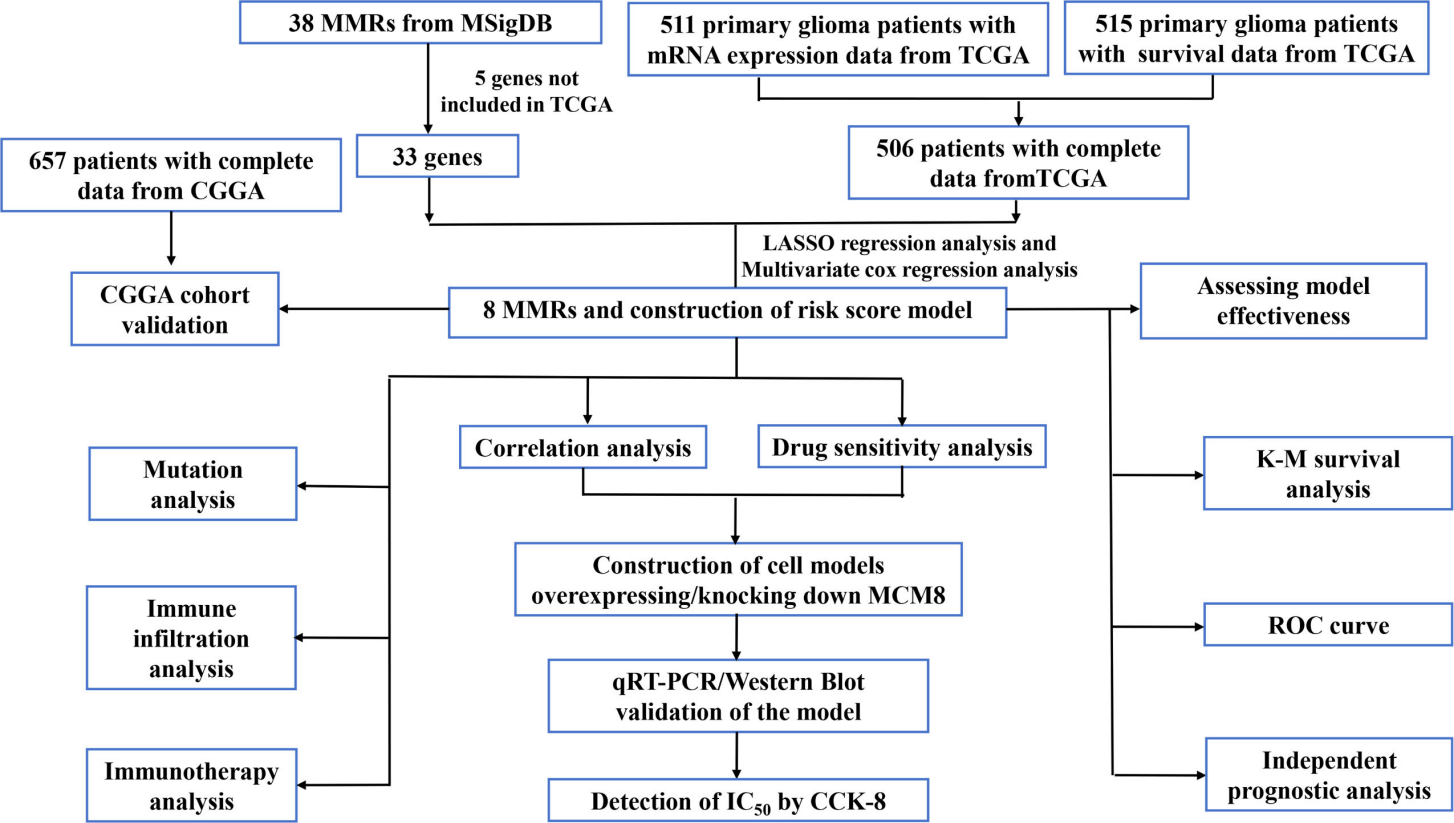
Flow chart of this study.

Flow of this study is shown in **Figure 1**. We obtained mRNA transcriptomic data and clinical information for 511 glioma patients from the TCGA database. After excluding cases with incomplete clinical or transcriptomic data, 506 patients with both complete datasets were retained. Furthermore, 657 patients with complete survival data were selected from the CGGA database to form the validation cohort.

**Figure 2:**
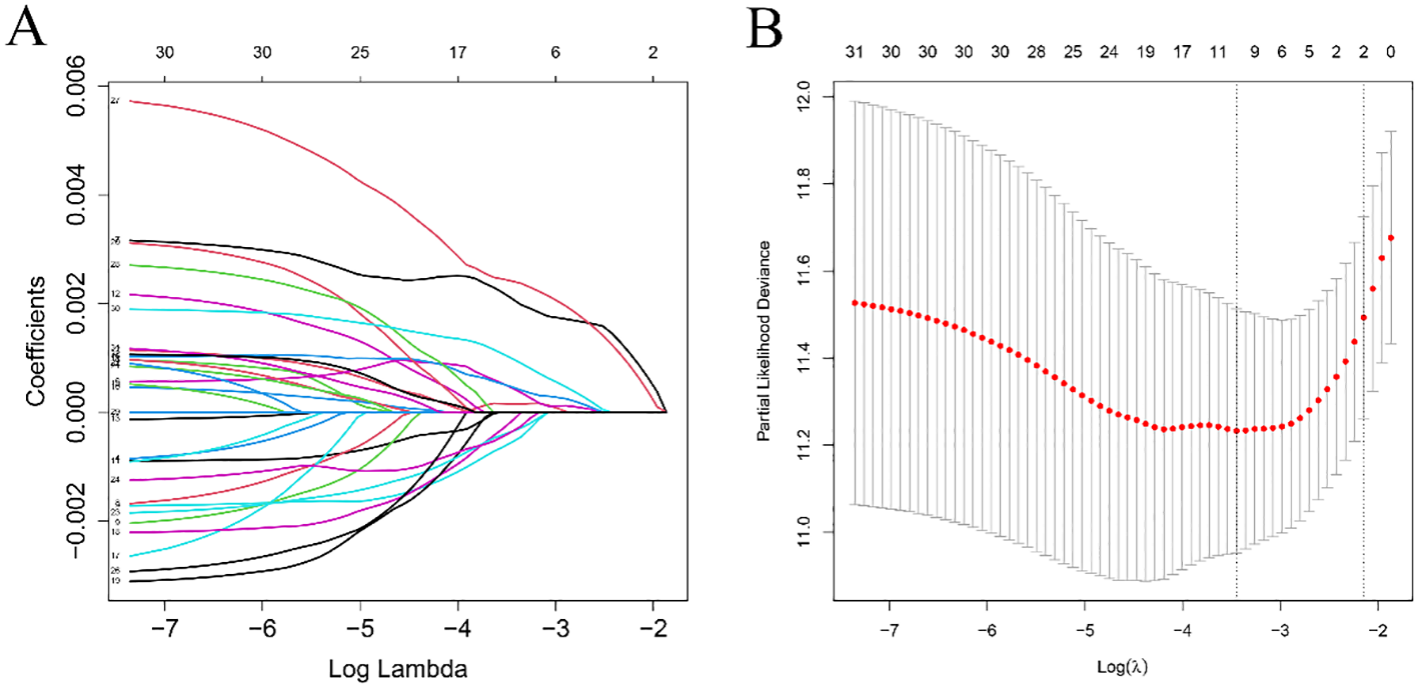
**(A)** LASSO regression model cvfit function. **(B)** LASSO regression modelling of lambda functions.

We systematically integrated the normalized mismatch repair-related genes with the newly released glioma transcriptome dataset from the TCGA database and its accompanying clinical information. Through machine learning algorithms, LASSO regression (**Figures 2A, B**) was first used to characterize MMR-related genes for dimensionality reduction, and then we used multivariate COX regression analysis to perform independent prognostic analysis of screening variables. After the dual screening strategy, eight genes closely associated with the prognosis were finally identified(HMGB1, MCM8, MUTYH, PMS1, RNASEH2B, RNASEH2C, RPA3, TP73)(**Table 2**). These eight genes exhibit diverse but interconnected functions within the DNA mismatch repair (MMR) and broader DNA damage response networks. Specifically, PMS1 is a core MMR component of the MutL homolog family, directly involved in mismatch recognition and repair. MUTYH functions in base excision repair (BER) to correct oxidative DNA damage. RPA3 is a subunit of the replication protein A (RPA) complex, essential for DNA replication, repair, and recombination. RNASEH2B and RNASEH2C are components of the RNase H2 complex, which removes RNA primers and misincorporated ribonucleotides from DNA. HMGB1 is a chromatin-binding protein that modulates DNA repair accessibility. MCM8 participates in homologous recombination repair and replication fork stability. TP73, a member of the p53 family, plays roles in DNA damage response and apoptosis. The integration of these functionally diverse yet MMR-associated genes into a single prognostic model reflects the complexity of genomic instability mechanisms in glioma.

**Table 2:** Details of the 8 genes are included in the signature.

mRNA	Ensemble ID	Chromosome location	β(Cox)	HR (95%CI)	P
HMGB1	ENSG00000189403	Chr13:30,456,704-30,617,597	-0.543	0.58 (0.32-1.05)	0.070
MCM8	ENSG00000125885	Chr20:5,950,652-5,998,977	0.819	2.27 (1.46-3.51)	<0.001
MUTYH	ENSG00000132781	Chr1:45,329,163-45,340,893	0.514	1.67 (1.06-2.64)	0.027
PMS1	ENSG00000064933	Chr2:189,784,085-189,877,629	-0.661	0.52 (0.28-0.94)	0.032
RNASEH2B	ENSG00000136104	Chr13:50,909,747-51,024,120	-0.575	0.56 (0.31-1.01)	0.055
RNASEH2C	ENSG00000172922	Chr11:65,714,005-65,720,818	-0.393	0.68 (0.42-1.08)	0.101
RPA3	ENSG00000106399	Chr7:7,636,518-7,718,607	0.496	1.64 (1.09-2.47)	0.019
TP73	ENSG00000078900	Chr1:3,652,516-3,736,201	0.296	1.34 (1.07-1.68)	0.010

### 3.2 Construction and validation of a set of eight predictive gene signatures for glioma patients

**Figure 3:**
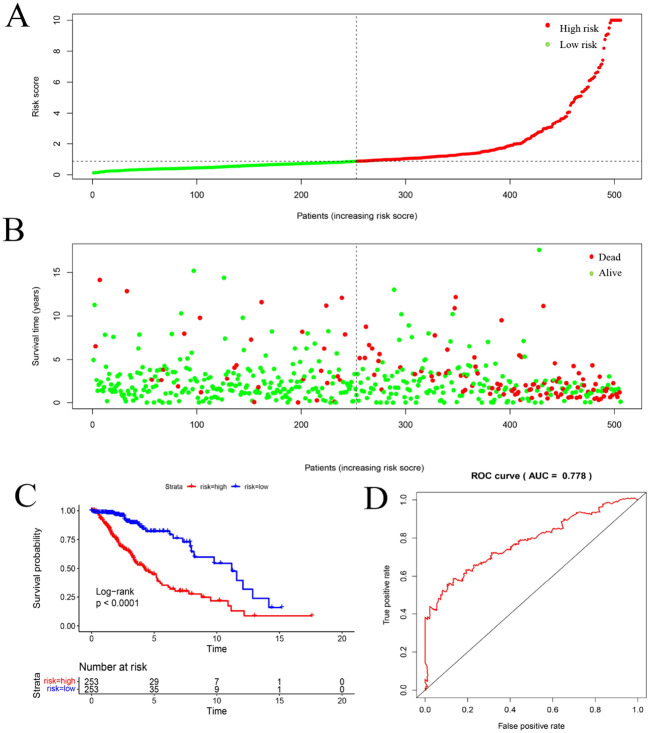
**(A)** Distribution of risk scores of each patient in TCGA. **(B)** Relationship between survival years and survival status of each patient in TCGA. **(C)** K-M curve to verify the predictive effect of the 8 genes signature in TCGA. **(D)** ROC curve analysis to evaluate the 5 years diagnostic efficacy of the 8 genes signature in TCGA.

Based on gene expression and coefficient of regression, we obtained a risk scoring system, namely the risk score: Risk Score = (-0.5431×HMGB1) + (0.8188×MCM8) + (0.5142×MUTYH) + (-0.6601×PMS1) + (-0.5753×RNASEH2B) + (-0.3929×RNASEH2C) + (0.4964×RPA3) + (0.2963×TP73). The risk scores were calculated according to the above formula for all patients in TCGA. Among them, the positive regression coefficient genes (MCM8, MUTYH, RPA3, TP73) were defined as risk promoters (HR>1), and the negative coefficient genes (HMGB1, PMS1, RNASEH2B, RNASEH2C) were categorized as risk suppressors (HR<1). All 506 patients were risk stratified by the median risk score, and the high-risk and low-risk groups each contained 253 cases, showing a completely symmetrical distribution (**Figure 3A**). Survival scatter plots based on risk scores showed that the probability of survival of patients gradually decreased with progressively higher risk scores (**Figure 3B**). Patients in the low-risk group had a considerably superior overall survival (OS) than those in the high group, according to Kaplan-Meier survival analysis (**Figure 3C**). Additionally, we used ROC curve analysis to evaluate the model’s diagnostic role. The findings revealed that the model’s AUC value was 0.778 (**Figure 3D**), suggesting that the prognostic model built with the 8 MMR-related genes had a high accuracy.

**Figure 4:**
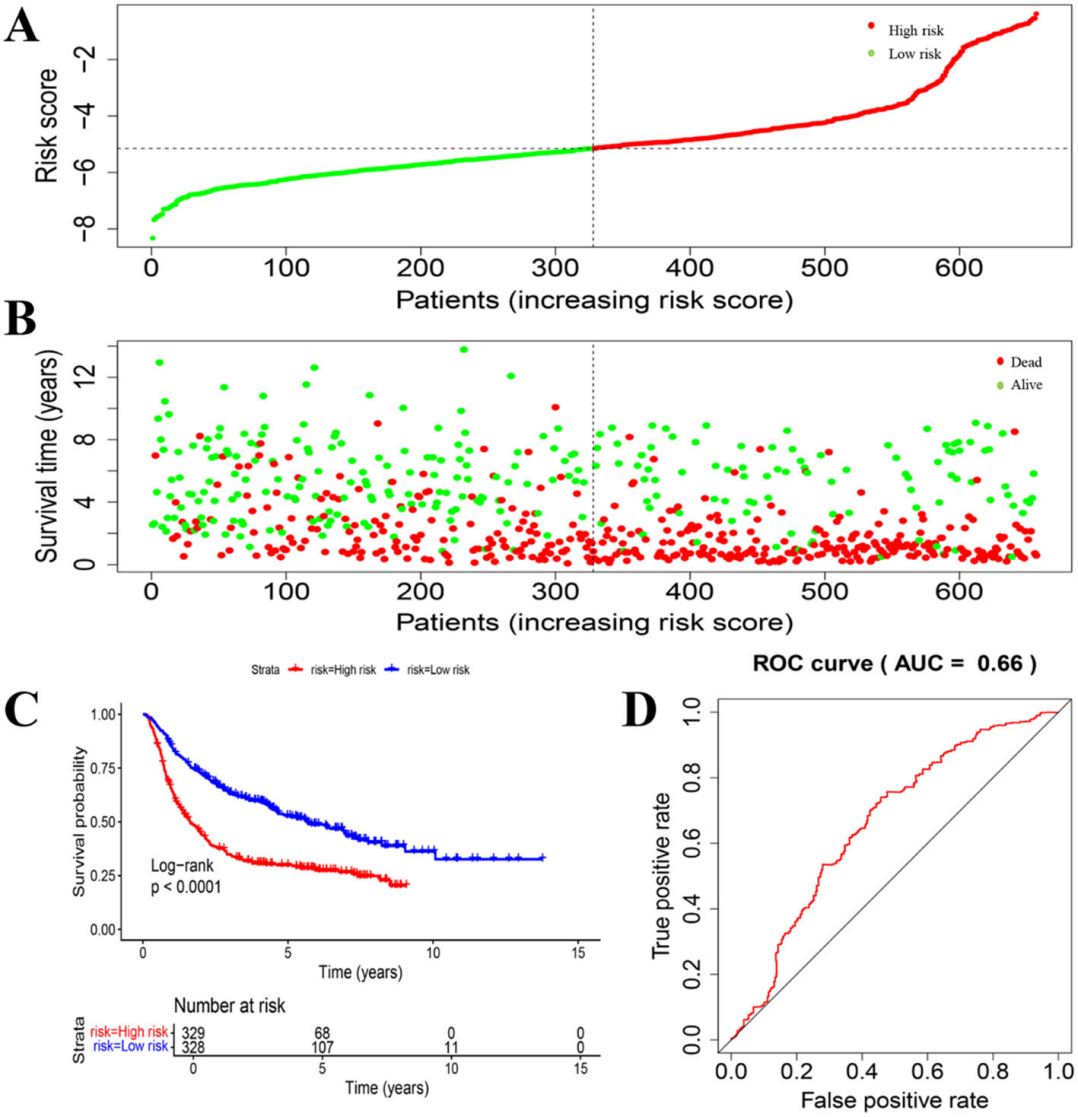
**(A)** Distribution of risk scores of each patient in CGGA. **(B)** Relationship between survival years and survival status of each patient in CGGA. **(C)** K-M curve to verify the predictive effect of the 8 genes signature in CGGA. **(D)** ROC curve analysis to evaluate the 5 years diagnostic efficacy of the 8 genes signature in CGGA.

To validate our risk scoring model, we applied the same gene coefficients and formula to the CGGA database, calculating a risk score for each patient and stratifying them into high- and low-risk groups based on the median score (**Figure 4A**). Consistent with the TCGA findings, most deaths were concentrated in the high-risk group (**Figure 4B**). Kaplan-Meier survival analysis further confirmed a higher mortality rate in the high-risk group within the CGGA dataset (**Figure 4C**). Moreover, ROC analysis yielded an AUC of 0.66 for the CGGA dataset. This further confirmed the model’s dependability and potential for clinical use.

### 3.3 Expression and genetic variation of 8 genes

**Figure 5:**
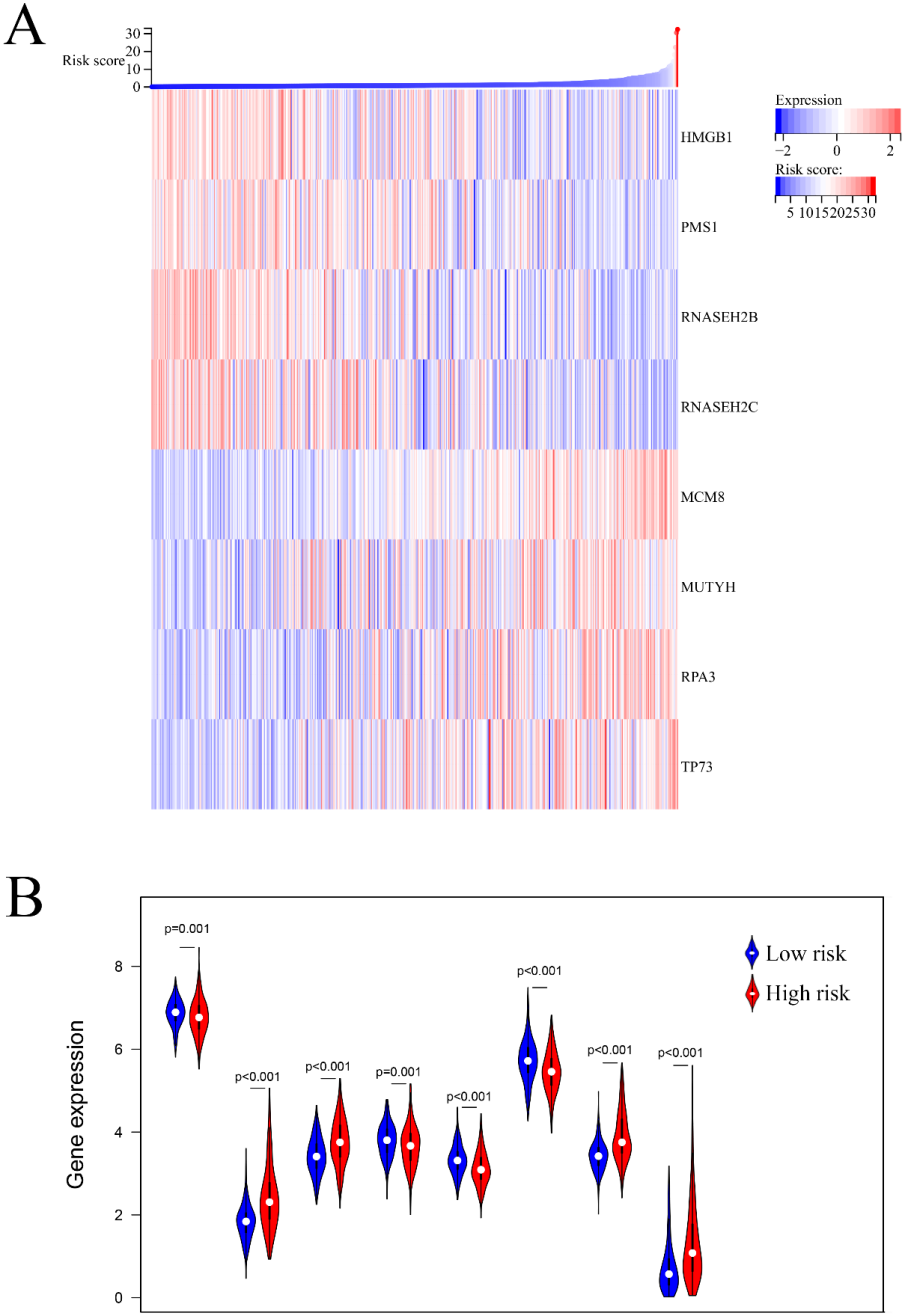
**(A)** The heat map for the expression of the 8 genes in glioma patients. **(B)** The expression of the 8 genes in low and high-risk group.

**Figure 6:**
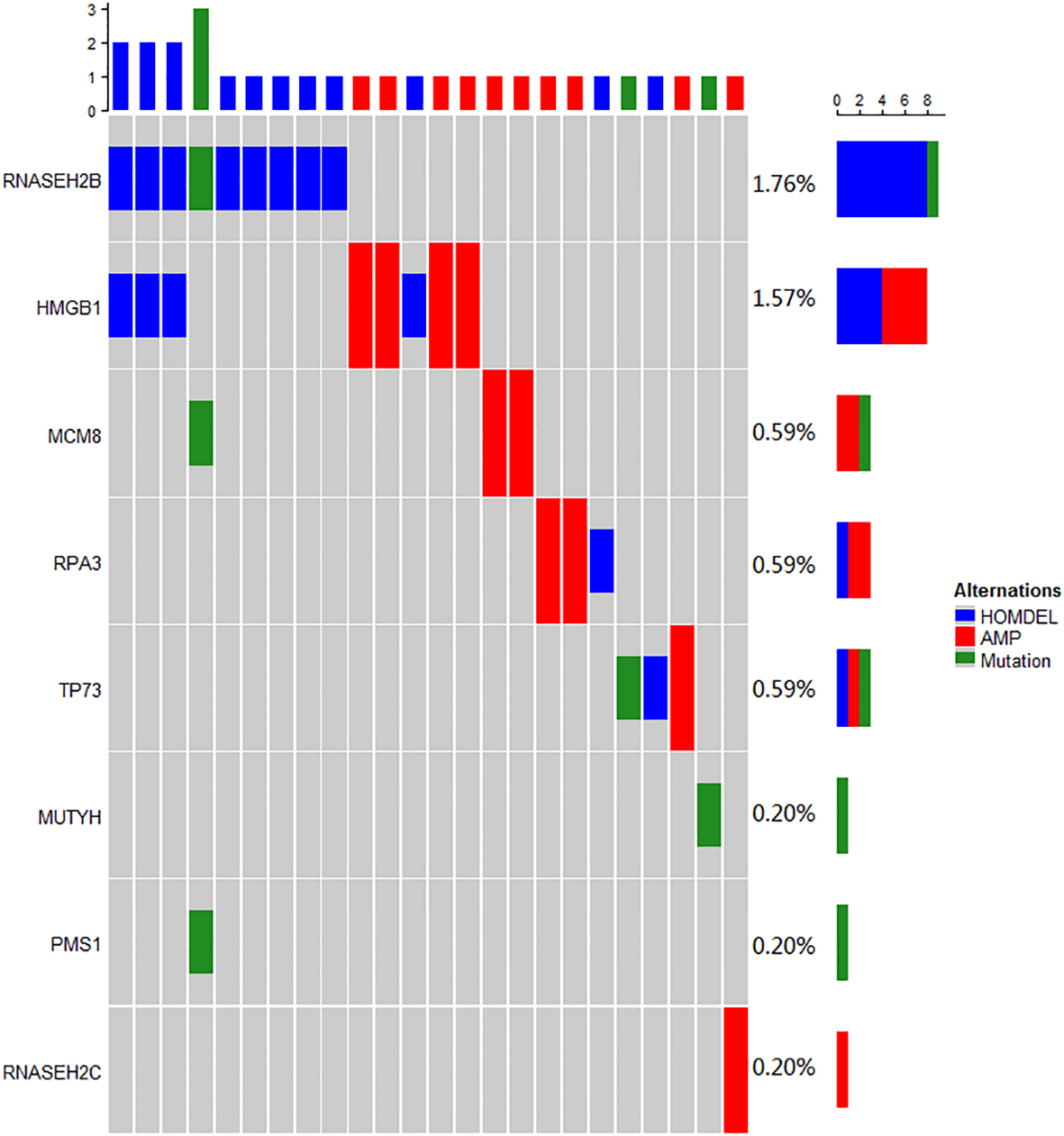
The mutation landscape of the 8 genes in glioma.

The eight genes in the model differed significantly in expression, with the expression of the protective genes HMGB1, PMS1, RNASEH2B, and RNASEH2C decreasing with increasing risk scores, whereas the expression of the risk genes MCM8, MUTYH, RPA3, and TP73 increased with increasing risk scores (**Figures 5A, B**). The cBioPortal database was used to analyze the genetic alterations of eight genes selected in the prognostic risk model. The results showed that 24 (4.7%) of the 511 glioma patients had mutations, including 3 (0.59%) mutations, 11 (2.15%) pure deletions, and 10 (1.96%) amplifications (**Table 3**). According to the waterfall plot, the eight genes’ mutation rates ranged from high to low were 1.76% (RNASEH2B), 1.57% (HMGB1), 0.59% (MCM8), 0.59% (RPA3), 0.59% (TP73), 0.20% (MUTYH), 0.20% (PMS1), 0.20% (RNASEH2C) (**Figure 6**). The specific mutations of the eight genes are shown in **Table 4**.

**Table 3:** Details of the 8 genes in different alterations.

Alteration	Number of cases	Frequency
HOMDEL	11	2.15%
Amplification	10	1.96%
Mutation	3	0.59%
Total	24	4.70%

**Table 4:** Details of the 8 genes in different alterations.

mRNAs	No alterations	Genetic alteration			Altered/profiled (%)
		HOMDEL	AMP	Mutation	
HMGB1	503	4	4	0	1.57%
MCM8	508	0	2	1	0.59%
MUTYH	510	0	0	1	0.20%
PMS1	510	0	0	1	0.20%
RNASEH2B	502	8	0	1	1.76%
RNASEH2C	510	0	1	0	0.20%
RPA3	508	1	2	0	0.59%
TP73	508	1	1	1	0.59%

### 3.4 Individual predictive value of the 8 genes signature

**Figure 7:**
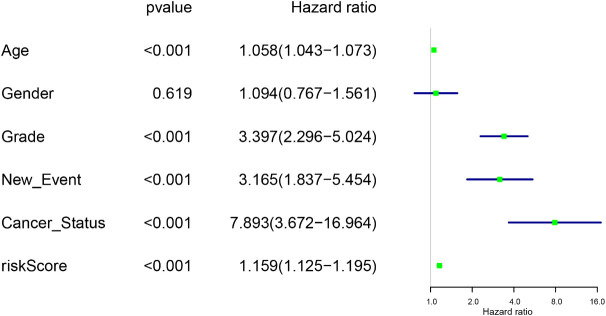
Univariable analyses for risk score and clinical features.

**Figure 8:**
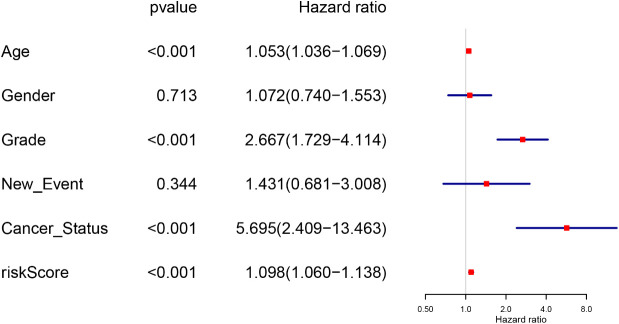
7 multivariable analyses for risk score and clinical features.

To assess differences in clinical parameters among patients with different risk scores, a chi-square test was performed (**Table 5**). The test results indicated that new events and tumor grades were significantly associated with patient risk scores. Subsequently, the risk score was integrated with clinical parameters such as age, grade, gender, new events and cancer status for univariate Cox regression analysis; furthermore, multivariate Cox regression analysis was performed to assess whether the eight-gene signature could serve as an independent prognostic predictor of survival outcomes in glioma patients. Univariate analysis confirmed that the 8-gene risk score is an independent prognostic indicator for patient survival, with a statistically significant association (HR = 1.159, 95% CI = 1.125-1.195, *p* < 0.001; **Figure 7**). After adjusting for confounding factors, the results of the multivariable Cox regression analysis still indicated that the risk score retained its property as an independent prognostic indicator (HR = 1.098, 95% CI = 1.060-1.138, *p* < 0.001; **Figure 8**). Additionally, the analysis suggested that age, grade, and cancer status remained independent prognostic factors (*p* < 0.05).

**Table 5:** The relation between risk score and clinical parameters.

Variables	Total (n=506)	High risk (n=253)	Low risk (n=253)	P
Age, Median (Q1, Q3)	41 (32.25, 53)	43 (32, 57)	39 (33, 49)	0.052
Gender, n (%)				0.788
Female	226 (45)	111 (44)	115 (45)	
Male	280 (55)	142 (56)	138 (55)	
Grade, n (%)				<0.001
2	245 (49)	88 (35)	157 (62)	
3	260 (51)	165 (65)	95 (38)	
New_Event, n (%)				0.004
NO	231 (46)	99 (39)	132 (52)	
YES	275 (54)	154 (61)	121 (48)	
Cancer_Status, n (%)				0.885
Tumor free	175 (39)	89 (39)	86 (40)	
With tumor	271 (61)	141 (61)	130 (60)	

### 3.5 The immune landscape of prognostic models

**Figure 9:**
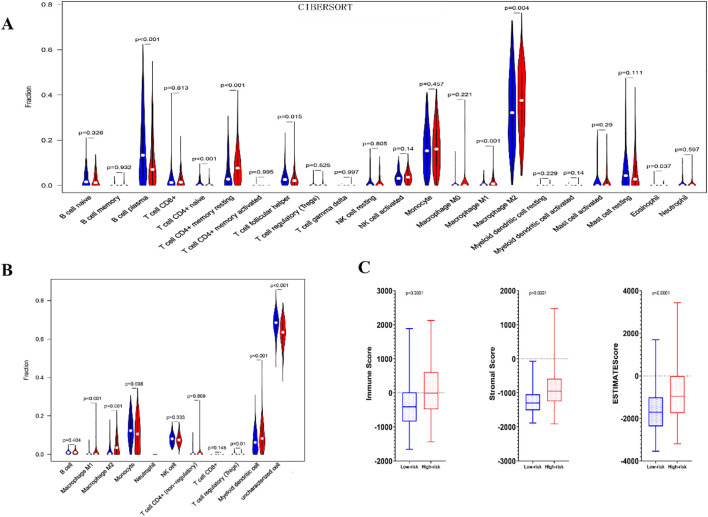
Immune cell infiltration landscape in high- and low-risk glioma patients. **(A, B)** Comparison of immune cell infiltration levels between high-risk and low-risk groups as assessed by CIBERSORT **(A)** and QUANTISEQ **(B)** algorithms. In the high-risk group, significant enrichment was observed for resting memory CD4+ T cells, M1-type macrophages (classically activated, pro-inflammatory and anti-tumor), and M2-type macrophages (alternatively activated, immunosuppressive and pro-tumor). The concurrent elevation of both M1 and M2 macrophages in the high-risk group reflects a complex tumor microenvironment with mixed inflammatory and suppressive features. In contrast, the low-risk group exhibited higher infiltration of plasma cells (terminally differentiated B lymphocytes mediating humoral immunity), follicular helper T cells (Tfh, known to express PD-1 and promote anti-tumor immune responses), and naive CD4+ T cells. **(C)** ESTIMATE algorithm analysis showing that the high-risk group had significantly higher Stromal Score, Immune Score, and ESTIMATE Score compared to the low-risk group (*P* < 0.0001), indicating increased infiltration of immune and stromal cells in the tumor microenvironment of high-risk patients, which may contribute to immune evasion and worse prognosis.

To analyze the immune microenvironment characteristics of different risk subgroups, this study combined the CIBERSORT and QUANTISEQ algorithms to assess the immune cell infiltration profiles of two patient groups. CIBERSORT analysis revealed that the microenvironment of low-risk patients was characterized by high levels of plasma cells, naive CD4+ T cells, follicular helper T cells, and Eosinophil. In contrast, the high-risk group exhibited significant enrichment of resting memory CD4+ T cells, Macrophage M1, and Macrophage M2. QUANTISEQ analysis further showed that the low-risk group had higher levels of infiltration by Monocyte, Tregs, and uncharacterized cells, whereas the high-risk group exhibited high infiltration of Myeloid dendritic cell, M1-type and M2-type macrophages (**Figures 9A, B**). Additionally, ESTIMATE algorithm calculations of microenvironment components revealed that the high-risk group had significantly higher ESTIMATE scores, stromal scores and immune scores than the low-risk group (*P* < 0.0001), indicating an increased abundance of infiltrating immune cells (*P* < 0.0001) (**Figure 9C**).

### 3.6 Immunotherapy and drug sensitivity analysis

**Figure 10:**
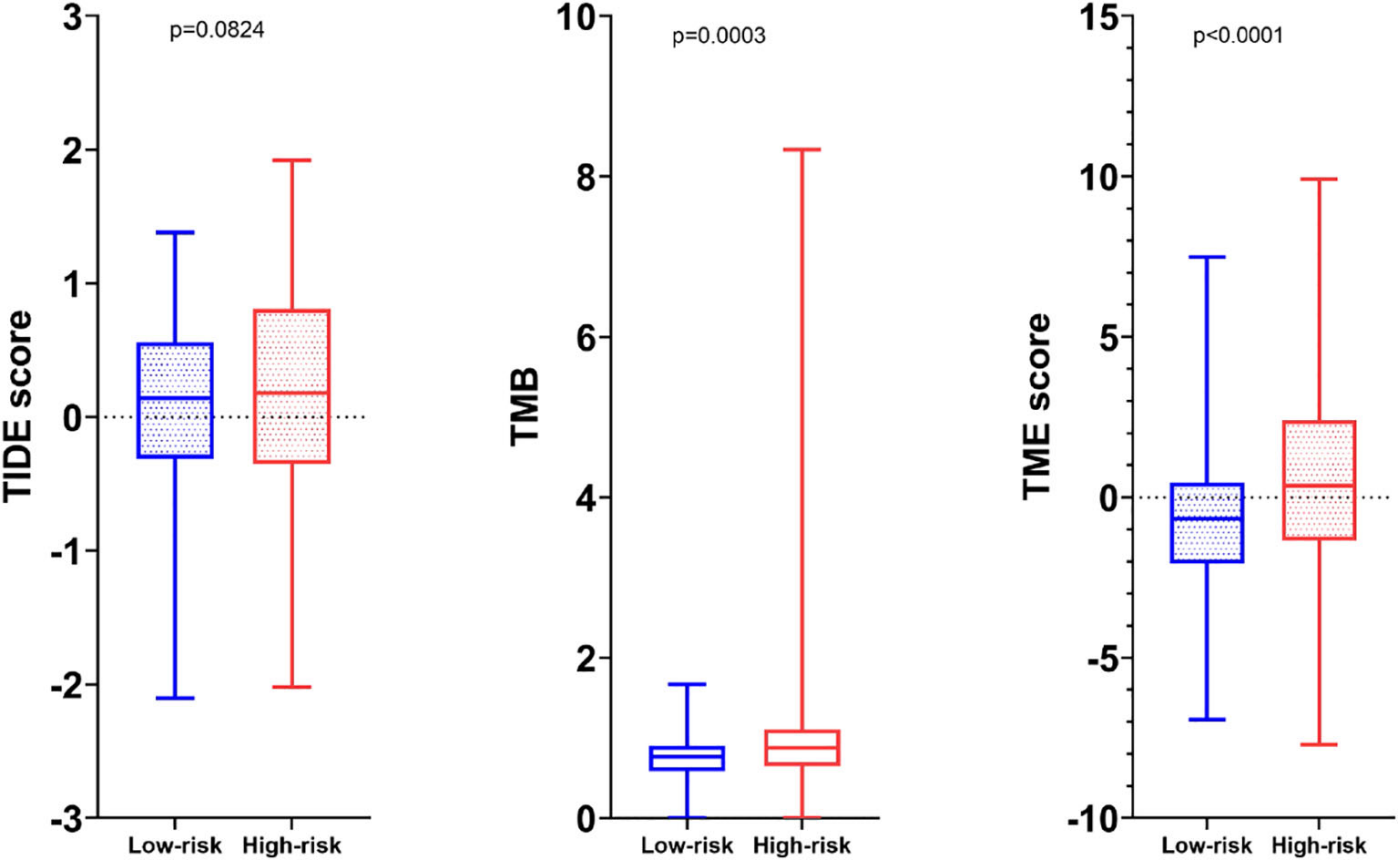
Immunotherapy and drug sensitivity analysis. TIDE score, TMB score and TME score in high and low risk groups.

To examine whether risk scores are significantly associated with immunotherapy outcomes, including TMB scores, TIDE scores, and TME scores. We found that TMB and TME scores were higher in the high-risk group, suggesting that patients in the high-risk group were more immune evasive and had relatively poorer immunotherapy outcomes (**Figure 10**).

**Figure 11:**
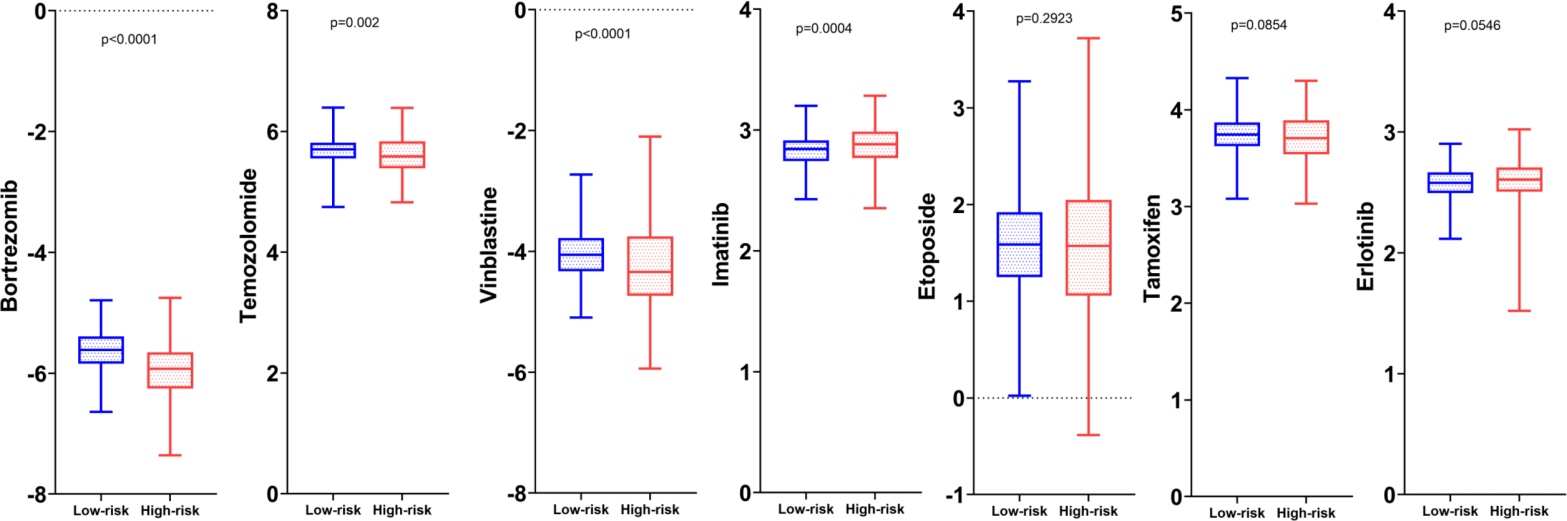
Differences in IC_50_ values among the seven chemotherapeutic agents in the high- and low-risk groups.

We compared the IC_50_ values of the two risk groups using the “pRRophetic” R package to analyze the differences in the sensitivity of potential chemotherapeutic agents for glioma. The findings demonstrated that the IC_50_ values of imatinib were significantly lower in the low-risk group and significantly lower in the high-risk group for bortezomib, TMZ, and vincristine (*P* < 0.05). This indicates higher sensitivity to imatinib at low risk and higher sensitivity to TMZ, bortezomib, and vincristine at high risk (**Figure 11**). These results suggest that the constructed prognostic model has the potential to be used as a biomarker for identifying the sensitivity to drug therapy and to inform the guidance of drug therapy for patients.

**Figure 12:**
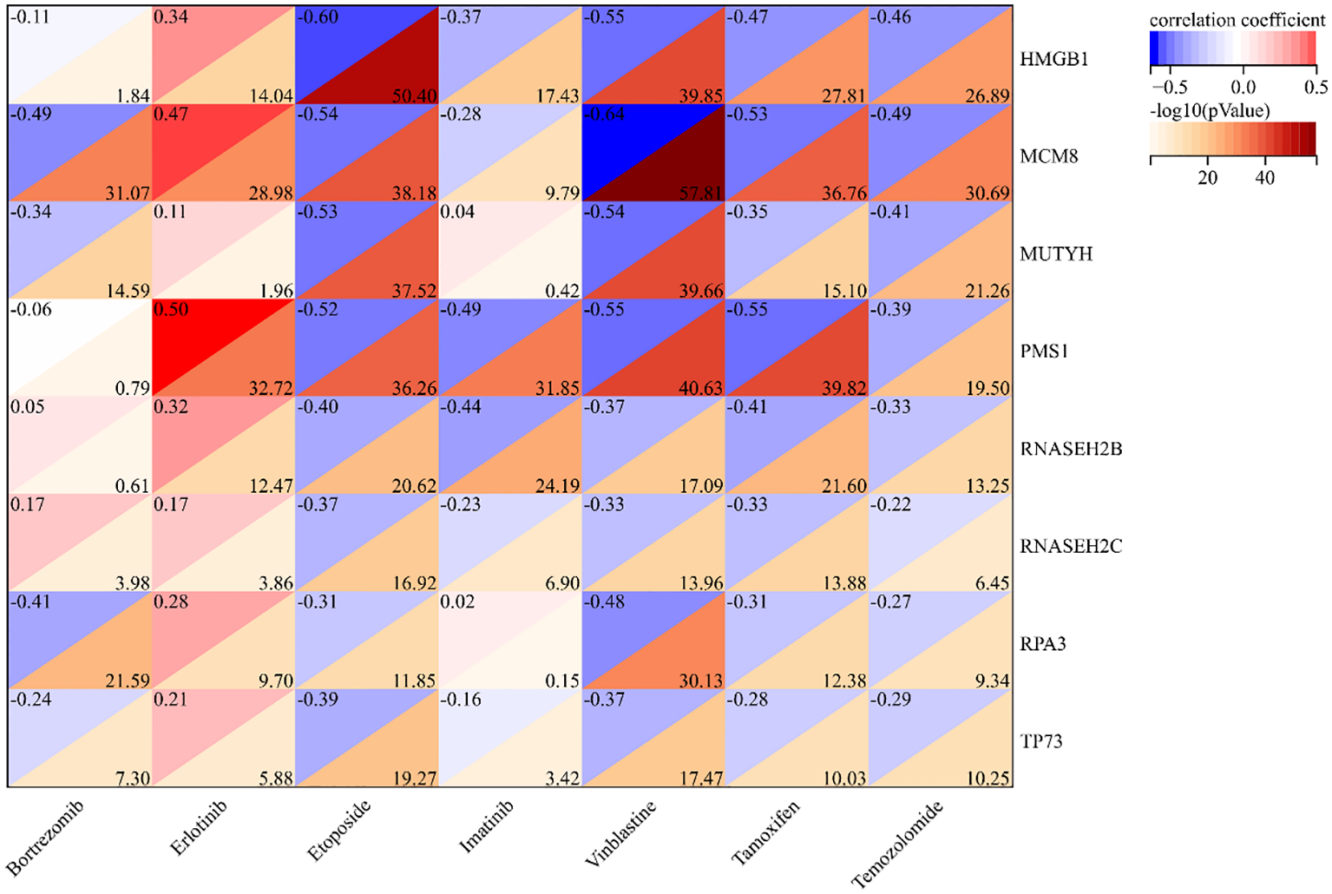
Correlation analysis of 7 chemotherapeutic agents and eight mismatch repair-related genes.

In addition, we analyzed the relationship between the eight risk genes included in the prognostic risk model and seven glioma chemotherapeutic agents. The analysis results revealed that the IC_50_ value of TMZ, a first-line chemotherapy drug for glioma, was negatively correlated with MCM8, and the correlation was the highest (**Figure 12**). Therefore, the MCM8 gene and TMZ were selected for further validation experiments of this study.

### 3.7 Cell models overexpressing and knocking down MCM8

**Figure 13:**
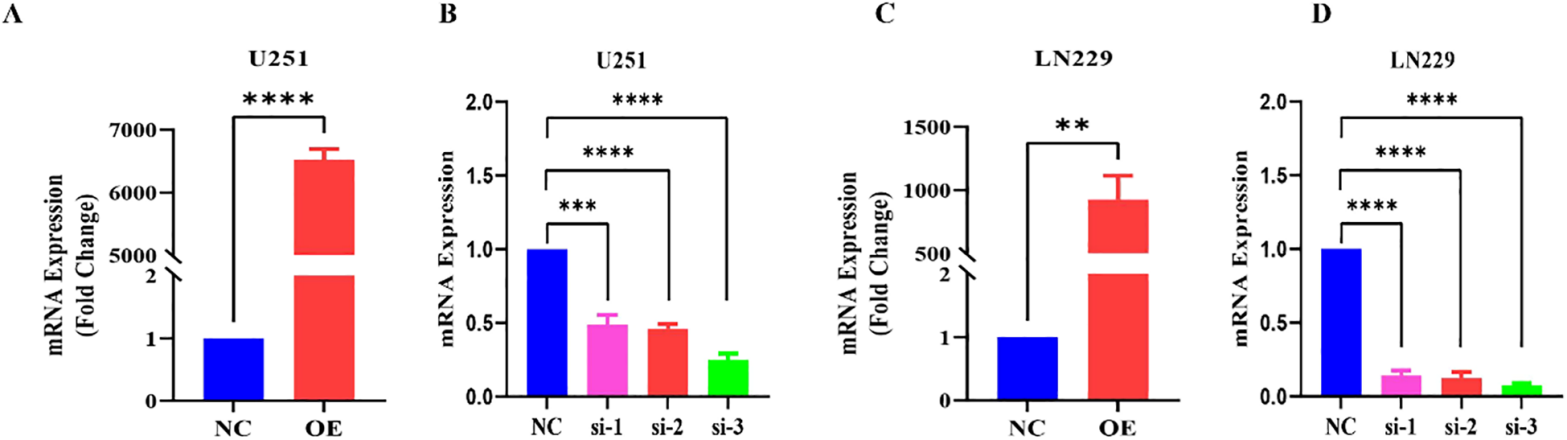
**(A–D)** qRT-PCR detection of MCM8 expression in U251 and LN229 cell line after overexpression/knockdown transfection.

**Figure 14:**
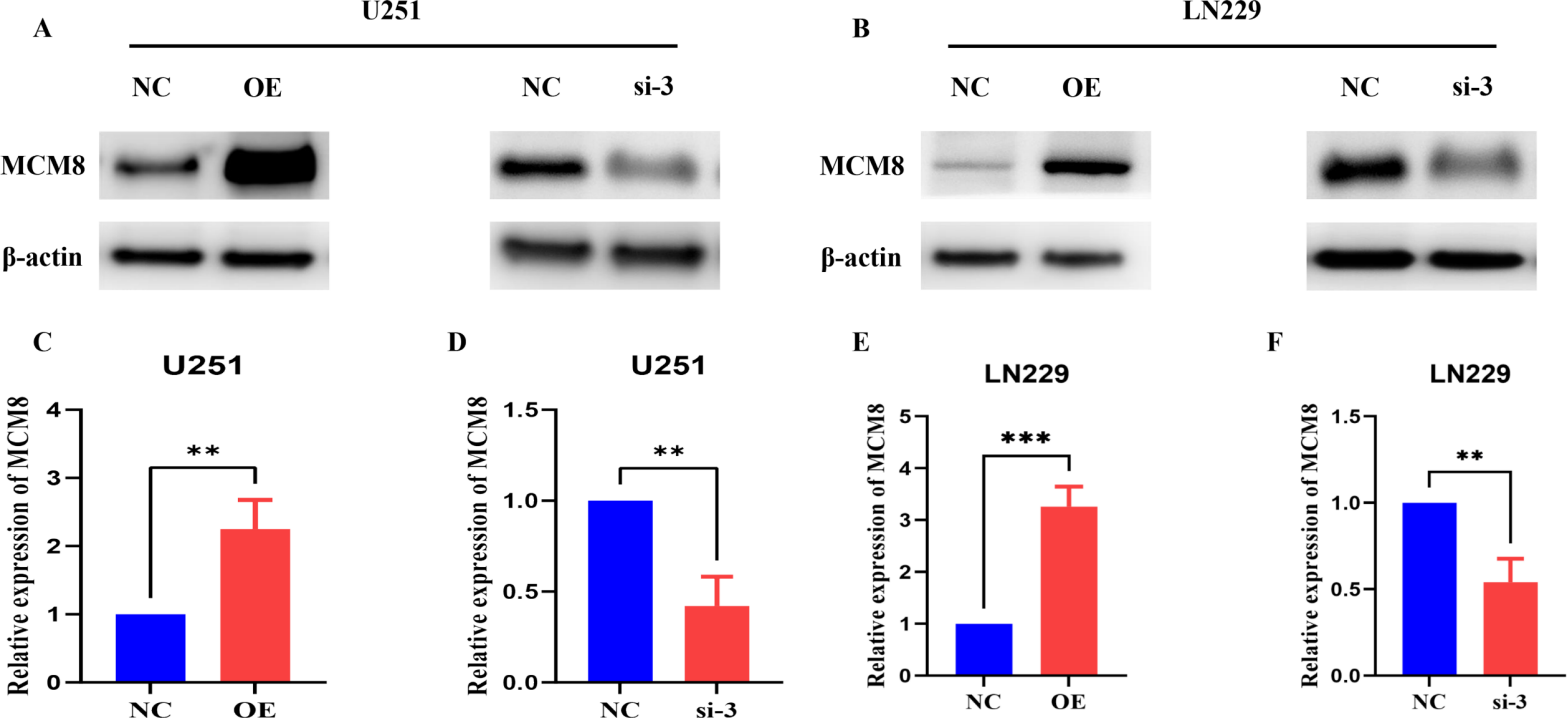
**(A, B)** Validation of overexpression and knockdown of MCM8 protein expression in U251/LN229 cell line. **(C–F)** Comparative histogram of proteins in the OE, NC, si-3 and NC in the U251 and LN229 cell line. (**P < 0.01, ***P < 0.001, ****P < 0.0001).

The qRT-PCR results showed that in U251 and LN229 cells, MCM8 mRNA levels in the overexpression group (OE) were significantly upregulated compared to the negative control group (NC) (**Figures 13A, C**). In contrast, MCM8 mRNA expression in the siRNA interference group was significantly lower than that in the NC group (**Figures 13B, D**), with the si-3 group showing the most effective gene silencing. Western blot protein quantification analysis further validated these results: MCM8 protein abundance in the OE group was significantly higher than that in the NC group, while the si-3 group showed a significant decrease in protein levels (**Figures 14A–F**). All experiments were performed in three independent biological replicates, and consistent results were observed across replicates (coefficient of variation< 10% for qRT-PCR measurements). The above data indicates that MCM8 overexpression and knockdown cell models were successfully established in U251 and LN229 cells. This model was subsequently applied to subsequent TMZ sensitivity experiments.

### 3.8 Drug sensitivity of TMZ affected by MCM8

**Figure 15:**
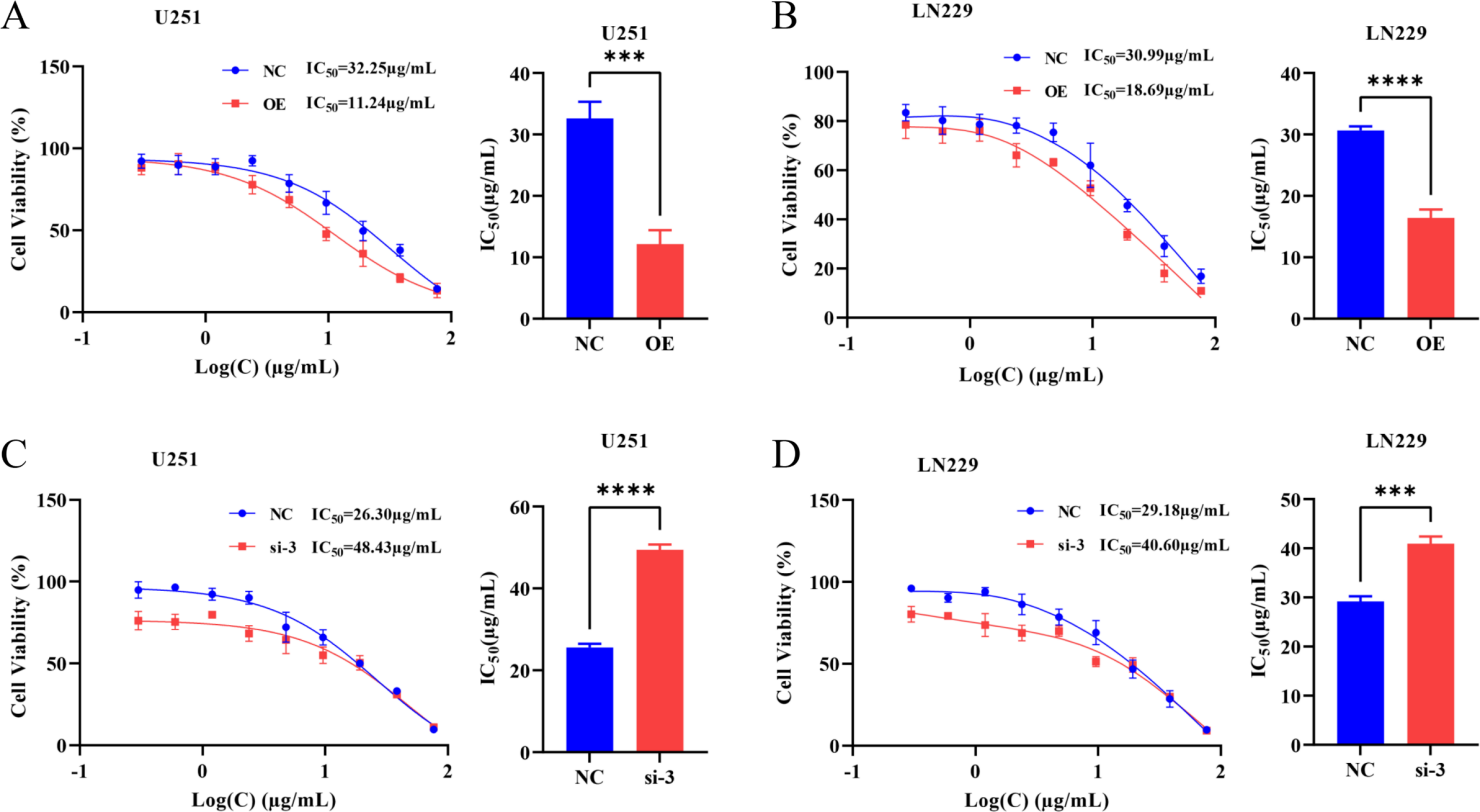
**(A, B)** Survival of U251/LN229 cell line in NC and OE groups after transfection. **(C, D)** Survival of U251/LN229 cell line in NC and si-3 groups after transfection. (***P < 0.001, ****P < 0.0001).

The MCM8 OE model was constructed by gene editing technology, and in U251 and LN229 cells, the IC_50_ values of the MCM8 overexpression group were significantly lower than NC group (**Figures 15A, B**), confirming that MCM8 gene overexpression effectively enhances the therapeutic sensitivity of glioma cells to TMZ. Conversely, in the reverse validation experiment, after knocking down MCM8 in the si-3 group, the IC_50_ values of both cell lines were significantly higher than those of the NC group (**Figures 15C, D**), indicating that MCM8 gene knockdown significantly weakened the therapeutic response of glioma cells to TMZ.

## 4 Discussion

This research employed advanced bioinformatics techniques to pinpoint eight key genes involved in MMR. Building on these findings, we developed a highly accurate prognostic risk assessment tool that effectively predicts survival outcomes for individuals with glioma. From a systems biology perspective, our approach aligns with recent multi-omics integration frameworks that jointly analyze genomic instability, immune microenvironment, and therapeutic response across cancers (23). We acknowledge that the research paradigm combining prognostic signature construction, immune microenvironment analysis, and drug sensitivity prediction has become increasingly prevalent in glioma bioinformatics. In this regard, our study does not claim to introduce a fundamentally new biological discovery but rather represents a “signature extension study” that applies this paradigm to mismatch repair-related genes.

A conceptually similar translational workflow-integrating large-scale transcriptomic discovery with downstream experimental validation-has been elegantly demonstrated in recent voltage-gated sodium channel (VGSC) research in glioma. The Jackson laboratory first performed pan-cancer VGSC analyses (24), subsequently identified SCN3B as a glioma-associated biomarker candidate (25), and further experimentally validated its functional relevance in modulating C6 glioma cell motility (26). These studies provide an exemplary methodological framework for our work. Our study follows a similar “discovery-to-validation” pipeline applied to MMR-related genes rather than VGSCs. This comparison does not diminish our contribution but rather situates it within a legitimate and reproducible scientific paradigm.

Specifically, our study extends these pan-cancer principles to glioma by demonstrating that an MMR-related gene signature can simultaneously stratify prognosis, characterize immune infiltration heterogeneity, and predict differential chemosensitivity-particularly TMZ response via MCM8 modulation. Furthermore, experimental validation revealed that MCM8, a key component of our model, modulates TMZ resistance in glioma U251 and LN229 cell lines. These research findings indicate MCM8 could be a viable therapeutic target in glioma.

Extensive research has highlighted the pivotal role of MMR in tumorigenesis and cancer progression (27), as well as its significant association with patient prognosis and radiotherapy sensitivity (28). Among the eight MMR genes included in our model, MCM8, MUTYH, RPA3 and TP73 are risk genes, while HMGB1, PMS1, RNASEH2B and RNASEH2C are protective genes. They may serve as innovative treatment targets and glioma outcome indicators. MCM8 expression correlated with worse glioma outcomes (29, 30). MUTYH mutations were associated with an increased risk of colorectal and ovarian cancers, among others (31). Elevated levels of MUTYH expression were an independent prognostic indicator in patients with low-grade gliomas (32). RPA3 promotes glioma proliferation, migration, and invasion through activation of the PI3K-AKT-mTOR pathway, with elevated expression in tumors linked to worse clinical outcomes (33). High expression of TP73 is independently associated with poor prognosis in WHO grade II/III gliomas, rendering poor prognosis glioblastoma stem cells resistant to TMZ (34, 35). The reliability of the prognostic risk model in this investigation is further supported by the studies which are in line with our findings. High HMGB1 expression may be a prognostic factor and has been linked in one study to a bad prognosis for glioma patients (36). Defective PMS1 function may lead to microsatellite instability (MSI), which increases the accumulation of mutations and contributes to glioma development and progression (37), and some studies have suggested that PMS1 is associated with tumor susceptibility (38). Several studies have identified associations between RNASEH2 gene expression and various cancer types (39–41), but RNASEH2B and RNASEH2C have been rarely investigated in gliomas, with only Ulrike Beyer et al. suggesting that a rare partial loss-of-function variant of RNASEH2B increases the risk of gliomas (42). HMGB1, PMS1, RNASEH2B and RNASEH2C play a role in gliomas still need to be further explored and could serve as novel diagnostic and prognostic markers for gliomas and provide new inspirations for identifying therapeutic targets for gliomas.

The TME, composed of secreted factors, signaling molecules, non-tumor cells, and extracellular matrix components, has been recognized as a key regulatory factor in the initiation, progression, and invasion of brain tumors (43). Studies have confirmed that the TME is a major player in glioma development and immunotherapeutic susceptibility (44). An increasing number of immunotherapy approaches have been introduced for the treatment of gliomas, such as DC vaccines, CAR-T therapy, and ICI (45). Therefore, focusing on the TME of gliomas may provide approaches to immunotherapy in the future (43). The study observed that the stromal score, immune score, and ESTIMATE score were all higher in the high-risk group compared to the low-risk group. Macrophages perform a critical function in the microenvironment of gliomas, influence glioma progression by promoting tumor growth, invasion, immune escape and treatment resistance (46, 47). Macrophage infiltration has been shown to be a poor prognostic factor for survival in glioma patients (48). This study further revealed that the levels of M1 and M2 macrophage infiltration were significantly higher in high-risk patients than in low-risk patients. Suggesting that macrophages may be effective targets for immunotherapy against gliomas. T follicular helper cells have a typical high expression of PD-1, which promotes the immune response to tumors. Plasma cells are key effectors in humoral immunity response as effector cells of terminal differentiation of B lymphocytes. Single-cell sequencing data analysis reveals that the proportion of plasma cells in high-grade gliomas showed a negative correlation with patient survival (49). This is also consistent with our findings. TMB is commonly acknowledged as a predictor of immunotherapy success, and the TME score gauges a patient’s reaction to immune checkpoint blockers (50). The TME and TMB scores of the high-risk group in our prognostic model were much higher than those of the low-risk group, indicating that the high-risk group had a greater capacity for immunological escape and comparatively worse outcomes with immunotherapy. A recently proposed framework for systems immunology emphasizes that, to transition from descriptive immune profiling to precision immunotherapy targeting, we must systematically identify actionable immune checkpoints and signaling pathways (45). Our risk model supports this goal by revealing distinct immune landscapes between high- and low-risk groups. We propose that the differential enrichment of specific immune subsets—such as macrophages and plasma cells—may represent novel therapeutic targets. Future studies should investigate whether modulating these subsets or their associated pathways improves prognosis in high-risk glioma patients.

Finally, we conducted a chemotherapeutic drug sensitivity analysis, which showed that low-risk patients had higher sensitivity to imatinib, while high-risk patients had higher sensitivity to bortezomib, TMZ, and vincristine. Lin Zhou et al. demonstrated that DAZL upregulates MCM8 to promote cisplatin resistance in non-small cell lung cancer (51). Subsequent experiments confirmed that MCM8 may serve as a biomarker for assessing temozolomide resistance in glioma. These findings suggest that the model may provide important reference information for personalized treatment of patients with gliomas.

However, several limitations of this study should be acknowledged. First, our current model did not comprehensively integrate well-established molecular stratification factors in glioma, including IDH mutation status, 1p/19q co-deletion, and MGMT promoter methylation. These factors are known to profoundly influence both prognosis and TMZ sensitivity. We therefore recommend that future studies incorporate these molecular factors alongside our MMR-related genes to develop a more comprehensive stratification system. Second, the use of bulk RNA-seq data from TCGA introduces inherent biases related to sample composition (e.g., variable tumor purity) and technical processing (e.g., batch effects). These biases may affect differential expression calls, pathway enrichment results, and-importantly-immune deconvolution estimates (52, 53). Our immune infiltration analyses using CIBERSORT and QUANTISEQ should therefore be interpreted as relative differences between risk groups rather than precise absolute quantifications. We encourage readers to view these results as hypothesis-generating, future studies using single-cell RNA sequencing, spatial transcriptomics, and functional immune assays will be essential to validate and extend our findings. Third, the validity of the model in clinical applications could not be determined, and further experiments need to be designed to confirm how the MCM8 gene affects TMZ sensitivity through the mismatch repair pathway.

## 5 Conclusion

This study establishes a prognostic risk model for glioma based on eight MMR-related genes. The model effectively stratifies patients, predicts survival, reflects distinct immune microenvironments, predicts differential responses to chemotherapy/immunotherapy, and demonstrates independent prognostic value. Furthermore, MCM8 was validated as a potential biomarker for TMZ sensitivity, highlighting its therapeutic relevance. The exploration of small-molecule inhibitors or CRISPR-based gene editing strategies targeting MCM8 may represent another avenue for preclinical research.

## Data Availability

The original contributions presented in the study are included in the article/supplementary material. Further inquiries can be directed to the corresponding authors.
